# Mitochondrial dysfunction as a key player in aggravating periodontitis among diabetic patients: review of the current scope of knowledge

**DOI:** 10.1007/s00210-025-04025-x

**Published:** 2025-04-24

**Authors:** Al-Hassan Soliman Wadan, Ahmed Sherief Moshref, Abdullah Mohammed Emam, Youssef Gamal Bakry, Bushra Osama Khalil, Akhilanand Chaurasia, Reham A. H. Ibrahim, Tamer Badawy, Samah S. Mehanny

**Affiliations:** 1 Department of Oral Biology, Faculty of Dentistry, Galala University, Galala City, Suez Egypt; 2 Faculty of Dentistry, Galala University, Galala City, Suez Egypt; 3 Department of Oral Medicine and Radiology, King George’S Medical University, Lucknow, India; 4 Department of Oral Biology, Faculty of Dentistry, Cairo University, Cairo, Egypt

**Keywords:** Diabetes, Periodontitis, Cytokines, Mitochondrial dysfunction, Reactive Oxygen Species (ROS)

## Abstract

Periodontitis is a prevalent inflammatory disease that leads to significant periodontal tissue destruction and compromised dental health, with its severity exacerbated in individuals with Diabetes Mellitus (DM). This review explores the complex relationship between mitochondrial dysfunction and periodontitis in diabetic patients. Recent studies indicate that the excessive production of reactive oxygen species (ROS), primarily generated by dysfunctional mitochondrial electron transport chain (ETC) complexes, contributes to oxidative stress (OS) and subsequent periodontal tissue damage. The interplay between impaired mitochondrial biogenesis, apoptosis of periodontal cells, and ROS accumulation highlights a critical area of concern in understanding the pathophysiology of diabetic periodontitis. Furthermore, altered glycemic control due to inflammatory processes associated with periodontitis may perpetuate a cyclical detriment to oral and systemic health. This review aims to highlight the mechanistic roles of mitochondrial dysfunction in the aggravation of periodontitis among diabetic patients, emphasizing further research to identify potential therapeutic targets and improve treatment efficacy for this dual pathology.

## Introduction

Periodontitis is a widespread and chronic metabolic and inflammatory condition that impacts approximately half of the world's older population (Deng et al. 2024). Patients' conditions are marked by the gradual degradation of periodontal tissue structures, such as the gingiva, alveolar bone, and periodontal ligament, which can ultimately result in tooth loss if not adequately managed (Deng et al. 2024; Zhao et al. 2023). Progression of periodontitis can be accentuated and accelerated by various systemic and pathogenic factors, including Diabetes Mellitus (DM) (Sun et al. 2017). Notably, a longitudinal study spanning 20 years identified a 29% higher risk of developing periodontitis among male patients with diabetes compared to their non-diabetic affected counterparts (Jimenez et al. 2012). Moreover, treatments for periodontitis are less effective in diabetic people compared to non-diabetic ones (Joseph et al. 2017). This may be attributed to the principal mechanism explaining the impact of diabetes on periodontitis remaining largely unexplored (Joseph et al. 2017). Oxidative stress (OS) is a pathophysiological process contributing to diabetic periodontitis (Altıngöz et al. 2021; Bala et al. 2019; Cairo et al. 2022; Duarte et al. 2011; García-Hernández al et al. 2018; Govindaraj et al. 2021; Patil et al. 2016; Portes et al. 2021). It is represented by the excessive generation of high ROS resulting from an imbalanced pro-oxidant/antioxidant equilibrium (Black et al. 2020; Chen et al. 2019; Ghasemi-Dehnoo et al. 2020; Hsu et al. 2012; Lugrin et al. 2013; Portes et al. 2021; Wang et al. 2017a). Extensive periodontal tissue destruction has been strongly linked to increased ROS production and a reduced antioxidant defense system in individuals with diabetes (Deng et al. 2024; Pan et al. 2024). Based on these findings, antioxidant administration has been shown to prevent the development of experimental diabetic periodontitis effectively (Black 2022; Ghasemi-Dehnoo et al. 2020; Mizutani et al. 2021; Mo et al. 2013; Rahimi-Madiseh et al. 2016; Sekhar 2022; Thomas et al. 2014). Although the critical role of OS is well-recognized, there remains a notable lack of in vivo studies exploring its specific contribution to the progression of periodontitis in diabetic conditions (Sun et al. 2017).

Mitochondria are both a significant source and primary target of ROS. Excessive production of ROS induces oxidative damage to mitochondrial proteins, resulting in structural modifications and functional impairment. Within mitochondria, the ETC, particularly complexes I and III, acts as a primary generator and a significant target of ROS. This disruption of the ETC is pivotal in the onset and progression of mitochondrial dysfunction (Lustgarten et al. 2012). Dysfunctional regulation of ETC complexes has been identified during extensive OS and is recognized as a major contributor to mitochondrial dysfunction. This impairment disrupts the normal flow of electrons, accumulating ROS, further exacerbating mitochondrial damage and impairing cellular energy production (Ding et al. 2021; Musatov and Robinson 2012). Excessive ROS can directly damage mitochondria DNA (mtDNA) by inhibiting its transcription (Dong et al. 2023; Portes et al. 2021). Additionally, OS can impair mitochondrial biogenesis, a process closely linked to mtDNA copy number (Siddiqi et al. 2020).

Mitochondrial biogenesis, a process that enhances mitochondrial mass and copy number within cells, supports cellular energy demands and overall function (Wang et al. 2011). The mitochondrial physiological biogenesis is adapted and regulated by the peroxisome PGC-1α-NRF1/2-TFAM pathway, a key mechanism for maintaining mitochondrial quality control (Zhang et al. 2010). Nonetheless, whether these mitochondrial-associated molecular mechanisms directly influence the pathological progression of diabetes-associated periodontitis (DP) is yet to be determined. Moreover, mitochondria play a critical role as intracellular organelles in the apoptosis mechanism (Deng et al. 2024). Mitochondrial dysfunction induced by apoptotic stimuli can result in passive cell death due to impaired energy production. Despite the well-established association between diabetes and periodontal disease severity, the mechanistic role of mitochondrial dysfunction in diabetic periodontitis remains largely unexplored. In diabetic patients, increased apoptosis of fibroblasts and osteoblasts may contribute to delayed healing and the progression of periodontal disease (Deng et al. 2024; Olsen et al. 2017; Rosen et al. 2001). However, the involvement of mitochondrial dysfunction in diabetes-associated cell apoptosis within periodontitis requires further investigation. This review targets the potential and crucial role of impaired mitochondrial function in the accelerated progression and heightened severity of periodontitis often observed in diabetic patients. Additionally, periodontitis may affect glycemic control in healthy patients. By investigating this critical link, we aim to identify new therapeutic targets and pathways that could lead to more effective treatments for patients suffering from both conditions.

## The pathophysiology of diabetes mellitus

Diabetes mellitus comprises a heterogeneous group of metabolic disorders, primarily defined by persistent hyperglycemia due to deficiencies in insulin secretion, action, or a combination of both. The condition is broadly categorized into major types, including Type 1 diabetes (T1D) (Nongrum et al. 2022), Type 2 diabetes (T2D) (Nongrum et al. 2022), gestational diabetes mellitus (GDM) (Bunpeng et al 2022), and specific types of diabetes due to other causes. T1D, formerly known as insulin-dependent diabetes, is an auto-immune metabolic defect in which the immune system targets and destroys pancreatic β-cells, resulting in complete insulin deficiency (Roy et al. 2019). In contrast, gestational diabetes mellitus (GDM) (Furman et al. 2019) develops during pregnancy when insulin production is inadequate to overcome the insulin resistance induced by placental hormones (Genco and Borgnakke 2020).

T2D, representing 90–95% of all diabetes cases, is marked by a progressive reduction in β-cell insulin secretion, typically occurring in conjunction with insulin resistance (Mealey and Oates 2006). The pathogenesis of T2D arises from complex interactions between genetic predisposition and environmental factors, with mitochondrial dysfunction playing a pivotal role in its onset and progression. Emerging evidence highlights the critical influence of mitochondrial dysfunction on insulin resistance and β-cell failure, as mitochondria are integral to glucose metabolism and energy regulation. Also, mitochondrial dysfunction can impair insulin signaling pathways, leading to lipid and diacylglycerol accumulation, further aggravating insulin resistance. This connection highlights the potential of focusing on mitochondrial health as an innovative therapeutic approach for managing T2D (Feng et al. 2011).

Oxidative stress, an imbalance between ROS production and antioxidant defenses, has become a key factor in the pathogenesis of both T1D and T2D, with mitochondrial dysfunction acting as both a cause and a consequence of oxidative damage (Bhatti et al. 2022; Darenskaya et al. 2021; Abu Khadra et al. 2024; Ma et al. 2018). Increased glucose levels, a defining characteristic of diabetes, stimulate excessive production of mitochondrial ROS, which damages mitochondrial DNA and proteins. This triggers a self-reinforcing cycle of oxidative stress and cellular dysfunction, which accelerates the progression of both T1D and T2D (Dong et al. 2023; Portes et al. 2021). The complex relationship between mitochondrial function and diabetes is further exemplified in rare forms of diabetes caused by mutations in mitochondrial DNA, known as mitochondrial diabetes, which typically presents with other systemic manifestations (Deng et al. 2024). The management of different types of diabetes varies significantly, with T1D requiring lifelong insulin therapy, while T2D management may include lifestyle modifications, oral hypoglycemic agents, and/or insulin therapy depending on disease severity and progression (Sun et al. 2017).

Recent therapeutic approaches have begun to focus on targeting mitochondrial dysfunction and oxidative stress, with compounds such as coenzyme Q10, lipoic acid, and mitochondria-targeted antioxidants showing promise in preclinical and clinical studies (Fields et al. 2023). Understanding the complex interplay between mitochondrial function, oxidative stress, and glucose metabolism has opened new avenues for therapeutic intervention in diabetes management, with emerging research focusing on developing strategies to preserve mitochondrial function and reduce oxidative damage in diabetic patients (Nazarov et al. 2017; Oyewole and Birch-Machin 2015; Zinovkin et al. 2023). Diabetes is now understood as a diverse group of disorders, each characterized by distinct pathophysiological mechanisms, but all share common traits, including mitochondrial dysfunction and oxidative stress. This understanding has paved the way for more personalized and targeted therapeutic strategies to enhance outcomes for individuals affected by these conditions (Fields et al. 2023; Pickles et al. 2018).

## The role of mitochondria in the pathogenesis of periodontitis in the absence of DM

Mitochondria play a pivotal role in the pathogenesis of periodontitis, primarily through their involvement in oxidative stress, inflammation, and cellular dysfunction. Mitochondrial dysfunction is linked to various mechanisms in periodontitis, including exacerbating inflammatory responses and impaired tissue repair, particularly in diabetic patients where mitochondrial alterations lead to increased oxidative stress and apoptosis (Aral et al. 2021). Key proteins such as Mitofusin-1 and Mitofusin-2 are crucial for maintaining mitochondrial integrity and regulating inflammation; their decreased levels correlate with heightened oxidative stress and inflammation in periodontal tissues. Additionally, pathogens like Porphyromonas gingivalis induce mitochondrial dysfunction, disrupting metabolic states and promoting reactive oxygen species production, further aggravating periodontal inflammation. Recent studies also suggest a causal relationship between mitochondrial function and periodontitis, indicating that specific mitochondrial enzymes may serve as protective or risk factors for the disease (Kırmızıgül et al. 2024; Domon et al. 2009; Zhai et al. 2018).

## How diabetes mellitus aggravates the development of periodontitis

The pathophysiological mechanisms by which both T1D and T2D contribute to oxidative stress and ROS generation involve complex cellular and molecular pathways that ultimately lead to tissue damage and diabetic complications (Sun et al. 2017). In T1D, the autoimmune destruction of pancreatic β-cells triggers a cascade of metabolic disturbances that enhance oxidative stress, primarily through hyperglycemia-induced mitochondrial electron transport chain dysfunction (Yaribeygi et al. 2020). The persistent hyperglycemia observed in both T1D and T2D drives increased activity through the polyol pathway, converting glucose to sorbitol via aldose reductase. This process depletes NADPH, a vital cofactor for maintaining glutathione in its reduced antioxidant state (Tang et al. 2012). This process results in decreased cellular antioxidant capacity and increased vulnerability to oxidative damage (Tang et al. 2012). Hyperglycemia also drives the formation of AGEs through the non-enzymatic glycation of proteins, lipids, and nucleic acids. When AGEs bind to their receptors RAGE, they activate inflammatory pathways and stimulate further ROS production via NADPH oxidase (Petroianu 2022). The ETC plays a crucial role in oxidative stress associated with diabetes, as elevated glucose levels intensify the proton gradient across the inner mitochondrial membrane. This leads to partial oxygen reduction and superoxide production, predominantly at complexes I and III (Tang et al. 2012).

In T2D, insulin resistance compounds these effects by promoting free fatty acid oxidation, which increases electron flux through the ETC and enhances ROS production (Lien et al. 2021). The PKC pathway, activated by both hyperglycemia and increased free fatty acids in diabetes, further contributes to oxidative stress by stimulating NADPH oxidases and promoting inflammatory cytokine production (Jubaidi et al. 2022; Schmidt et al. 2010). Chronic inflammation, characteristic of both T1D and T2D, creates a feed-forward loop where inflammatory mediators stimulate ROS production, and increased oxidative stress promotes inflammatory responses through activation of NF-κB and other redox-sensitive transcription factors (Petroianu 2022). The hexosamine pathway, upregulated in diabetic conditions, contributes to ROS generation through increased O-GlcNAcylation of mitochondrial proteins, affecting their function and stability (Jubaidi et al. 2022; Lien et al. 2021; Petroianu 2022; Schmidt et al. 2010).

Moreover, diabetes-induced ER stress leads to calcium dysregulation and mitochondrial dysfunction, further exacerbating ROS production and oxidative damage (Liiv et al. 2024). The compromised antioxidant defense systems in both types of diabetes, including reduced activities of SOD, catalase, and glutathione peroxidase, diminish the cellular capacity to neutralize excess ROS (Lien et al. 2021; Ly et al. 2017; Panda et al. 2022; Proulx et al. 2021). T2D-specific mechanisms include adipose tissue dysfunction and lipotoxicity, which contribute to oxidative stress through increased fatty acid oxidation and mitochondrial dysfunction in various tissues, particularly skeletal muscle and liver (Cojocaru et al. 2023; Wang et al. 2023). The interconnection between oxidative stress and insulin resistance in T2D creates a vicious cycle where ROS-induced damage to insulin signaling proteins further impairs glucose uptake and metabolism, leading to more oxidative stress (Abel et al. 2024). Recent research has also highlighted the role of mitochondrial dynamics (fission and fusion) in diabetes-induced oxidative stress, with both T1D and T2D promoting excessive mitochondrial fission, leading to fragmented mitochondrial networks and increased ROS production (Darenskaya et al. 2021; Abu Khadra et al. 2024). The combined impact of these pathways results in oxidative damage to cellular macromolecules, including lipid peroxidation, protein carbonylation, and DNA alterations, which collectively play a critical role in the onset and progression of diabetic complications in both T1DM and T2DM (Zhang et al. 2023a, b, c).

## The bidirectional relationship between DM and periodontitis

Most studies examining the bidirectional relationship between DM and periodontitis focus on T2DM, given its higher global prevalence and later onset, factors that increase the likelihood of T2DM intersecting with periodontitis. Studies have consistently demonstrated that elevated glycosylated hemoglobin levels are linked to an increased risk of periodontal damage and greater severity of periodontitis. While the relationship is not strictly linear, higher levels of hyperglycemia are associated with a heightened risk of periodontitis and eventual tooth loss. Recent research has shown that T2DM raises the prevalence of periodontitis by 34%, with T2DM patients experiencing a 0.89-mm more significant clinical attachment loss, 0.61-mm deeper periodontal pockets, 2.01 fewer surviving teeth, and 2.22 more lost teeth compared to individuals without diabetes (Wu et al. 2020). The two conditions mutually exacerbate each other's incidence and severity (Abu Khadra et al. 2024). A study conducted on an Italian population identified several factors, including a family history of T2DM, poor glycemic control, and elevated CRP levels, as significant predictors of severe periodontitis onset and progression. An increase of 1 unit in serum HbA1c was associated with a 60% increased probability of severe periodontitis, with T2DM patients exhibiting a higher risk when baseline glycosylated hemoglobin levels were elevated. These findings highlight the critical role of hyperglycemia duration, onset, and severity in the development of chronic periodontitis. Hyperglycemia enhances the inflammatory response in periodontal tissues, directly or indirectly activating AGEs and their receptor RAGE, which affect leukocyte and fibroblast functions, promote proinflammatory cytokine expression, and increase the RANKL/OPG ratio, thereby fostering osteoclast formation and alveolar bone resorption. Both cross-sectional and longitudinal studies have identified T2DM as a significant risk factor and a potential contributor to the development of periodontitis (Zhang et al. 2023a, b, c).

Although fewer clinical studies have investigated the relationship between T1DM and periodontitis, there are considerable variabilities in the methods used to assess clinical periodontal status. Most studies on T1DM focus on children or adolescents, leading to less definitive evidence regarding the relationship between periodontitis and T1DM compared to T2DM (Buduneli 2022). Sanz et al. (2018) mentioned insufficient evidence linking poor glycemic control with periodontitis in T1DM patients (Sanz et al. 2018). In contrast, other researchers showed that T2DM patients had a more fantastic CAL than T1DM patients (Roy et al. 2019). Despite this, studies on children with T1DM consistently report a higher incidence of periodontal disease compared to healthy children, suggesting a bidirectional relationship similar to that of T2DM and periodontitis (Cairo et al. 2022). A systematic review and meta-analysis revealed that individuals with T1DM had significantly poorer periodontal health, exhibiting higher plaque indices, deeper pocket depths, more bleeding on probing (BOP), and more fantastic CAL compared to non-diabetic children and adolescents (Jensenet al. 2020). Other studies confirmed that T1DM patients had poorer periodontal health, higher plaque scores, and more severe periodontal destruction than systemically healthy individuals (Ismail et al. 2015). A recent systematic review analyzing 11 studies on the subject further confirmed that T1DM is a significant risk factor for the development of periodontitis (Dicembrini et al. 2020). The prevalence of periodontitis among T1DM patients is more than double that of nondiabetic individuals, with the condition also being more severe (Taylor et al. 2013; Vomhof-DeKrey and Picklo Sr 2012). Notably, there is considerable variation in periodontal health depending on the state of glycemic control (Cairo et al. 2022). Additionally, poor glycemic control in children and adolescents with T1DM is associated with worsening periodontal health (Ismail et al. 2015). In comparison with healthy children, those with T1DM exhibit a significantly lower saliva secretion rate (Mahalakshmi et al. 2019), and the reduction in human β-defensin (HβD)−3 levels in their saliva may help explain their increased susceptibility to periodontal disease (Yilmaz et al. 2022).

## Molecular mechanisms involved in periodontitis-associated with DM

Recent studies have highlighted the molecular mechanisms linking diabetes and periodontitis, including OS (Sun et al. 2017), the microbiome (Ebersole et al. 2021; Lu et al. 2022; Xiao et al. 2017), complex inflammatory cytokines (Abusleme and Moutsopoulos 2016), AGE/RAGE (Nonaka et al. 2017; Plemmenos and Piperi 2022), adipokines, microRNA, host immune factors (Deng et al. 2024; Chen et al. 2019), alveolar bone resorption damage (Huang et al. 2020; Xiao et al. 2017), and epigenetic alterations (Ferioli et al. 2019; Li et al. 2021).

### Oxidative stresses (OS)

Oxidative stress occurs when there is an imbalance between oxidants and antioxidants, disrupting redox signaling and regulation, which can lead to molecular damage. This imbalance is closely linked to the pathogenesis of chronic metabolic disorders, such as diabetes mellitus, as well as chronic inflammatory diseases, including periodontal diseases. Disruptions in redox balance lead to cellular dysfunction, creating a prooxidant environment that produces excessive ROS, while the body's capacity to neutralize free radicals becomes impaired (Gyurko et al. 2006; Rahimi-Madiseh et al. 2016; Vincent 2018; Zhang et al. 2021). Defense systems include antioxidant systems such as CAT (Chen et al 2018; Marcelo et al. 2011; Patil et al. 2016; Sima et al. 2016), glutathione peroxidase (GPx) (Chen et al. 2019; Marcelo et al. 2011; Karsiyaka Hendek et al. 2014), and Cu–Zn SOD (Chen et al. 2019; Marcelo et al. 2011; Radović et al. 2018; Thomas et al. 2014), and vitamins like vitamin E, A, and C (Elenkova et al. 2018; Toraman et al. 2020) Play a vibrant role in mitigating this imbalance. OS is a key factor in the onset and progression of both DM and periodontitis, primarily through the upregulation of host immune and inflammatory responses and the subsequent damage to essential biomolecules, including proteins, lipids, and DNA. In diabetes, immune defense mechanisms are impaired, making it difficult to respond to elevated levels of subgingival pathogens in periodontitis, thus exacerbating periodontal tissue destruction. Simultaneous prooxidant conditions in periodontal tissue can impair insulin sensitivity, promote insulin resistance, and lead to systemic effects. The coexistence of diabetes and periodontitis may exacerbate this disruption of redox balance, intensifying oxidative stress and contributing to more severe pathological outcomes (Chiu et al. 2016; Elenkova et al. 2018; Pu et al. 2018).

### OS in DM(Fig. 1)

**Fig. 1:**
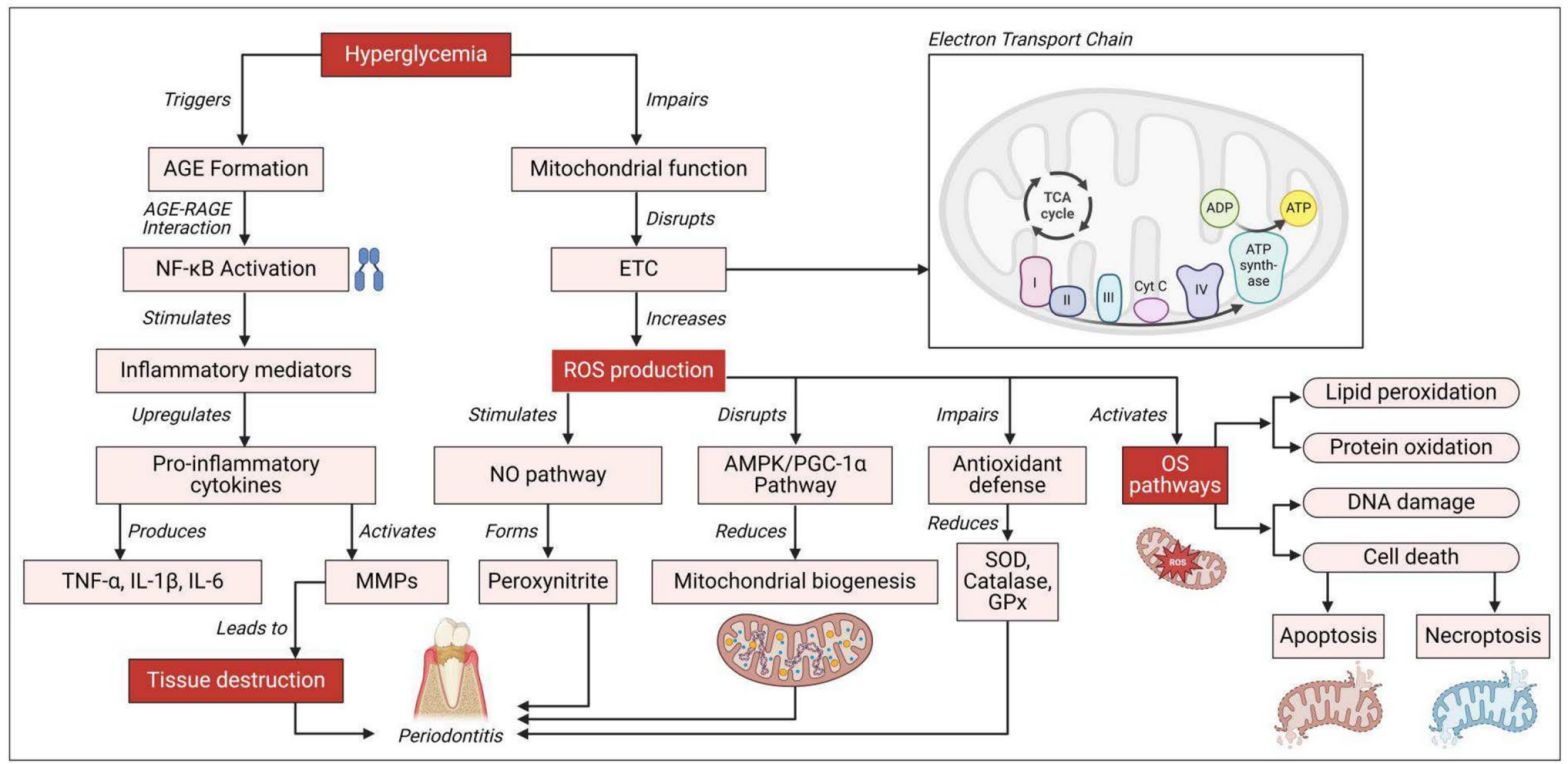
The figure outlines the complex molecular mechanisms contributing to oxidative stress and mitochondrial dysfunction in DP. Key molecular pathways drive these processes, including the AGE/RAGE axis. Hyperglycemia promotes the formation of AGEs, which interact with their receptor RAGE, activating NF-κB signaling and NADPH oxidase. This interaction leads to increased mitochondrial ROS production and the release of pro-inflammatory cytokines such as TNF-α, IL-1β, and IL-6. Simultaneously, dysfunction in mitochondrial ETC complexes, particularly complexes I and III, exacerbates superoxide (O2•-) generation, impairs ATP synthesis, and disrupts mitochondrial membrane potential. This results in the dysregulation of the AMPK/PGC-1α pathway, with decreased AMPK activation and reduced PGC-1α expression, leading to compromised mitochondrial biogenesis and antioxidant defenses. Oxidative stress induces lipid peroxidation, elevating markers such as malondialdehyde (MDA) and 4-hydroxynonenal (4-HNE), which damage cell membranes. Protein oxidation alters enzyme functions and accelerates protein degradation, while DNA damage, including mtDNA mutations and increased 8-hydroxy-2'-deoxyguanosine (8-OHdG) levels, impairs DNA repair mechanisms and triggers apoptosis. Elevated levels of pro-inflammatory cytokines (TNF-α, IL-1β, IL-6) and MMPs, particularly MMP-8 and MMP-9, promote extracellular matrix degradation, hampering periodontal tissue healing. The NO pathway is also implicated, with increased inducible nitric oxide synthase (iNOS) expression and nitric oxide production leading to peroxynitrite formation and nitrosative stress. Antioxidant defenses are severely compromised, as enzymatic antioxidants (SOD, catalase, GPx, and peroxiredoxin) are reduced, and non-enzymatic antioxidants (glutathione, vitamins C and E, coenzyme Q10) are depleted, impairing the body's ability to combat oxidative stress. Cell death pathways, including apoptosis and necroptosis, are activated, with enhanced cytochrome c release, caspase cascade activation, and a disrupted Bax/Bcl-2 ratio promoting apoptosis. The RIPK1/RIPK3/MLKL pathway also contributes to necroptosis, leading to irreversible tissue damage. Metabolic dysfunction, including impaired glucose metabolism and reduced tricarboxylic acid (TCA) cycle activity, compromises energy production, while dysregulated fatty acid metabolism leads to lipid accumulation and increased lipotoxicity. Tissue repair and regeneration are hindered due to the downregulation of growth factors like VEGF, PDGF, and TGF-β, reduced stem cell proliferation and differentiation capacity, and increased cellular senescence. These interconnected pathways illustrate the complexity of oxidative stress and mitochondrial dysfunction in diabetic periodontitis, emphasizing potential targets for therapeutic intervention, biomarker discovery, and personalized treatment approaches. Understanding these mechanisms is crucial for developing effective strategies to slow disease progression and reduce complications

The generation of ROS and the ensuing oxidative stress are central to the pathogenesis of DM. These processes are intricately associated with disrupted glucose metabolism, inadequate insulin secretion, and insulin resistance, collectively driving persistent hyperglycemia (Mealey and Oates 2006). Research has shown that oxidative stress markers, such as lipid peroxidation products (e.g., MDA) (Chen et al. 2019; Chen et al 2018), protein oxidation products, and DNA oxidation markers are significantly elevated in DM, accompanied by reduced activity of both enzymatic antioxidants and nonenzymatic antioxidants (Kocher et al 2018). Excessive ROS production arises from various mechanisms, including the polyol pathway (Black 2022; Ghasemi-Dehnoo et al. 2020; Munhoz et al. 2007), hexosamine (Ghasemi-Dehnoo et al. 2020), PKC (Karima et al. 2005), and other pathways (Elenkova et al. 2018). The excessive activation of these pathways results in a significant rise in intracellular oxidative stress. Hyperglycemia-induced reactive oxygen species production facilitates the polarization of M1-type proinflammatory macrophages, which are attracted to the inflammatory region where neutrophil-mediated respiratory bursts transpire. This mechanism releases supplementary proinflammatory mediators, further increasing ROS levels and intensifying oxidative stress (Alshial et al. 2023; Madkour et al. 2024; Soliman Wadan et al. 2024). The degree of oxidative stress is directly associated with the severity of inflammatory responses and mitochondrial dysfunction, which hinder insulin secretion and intensify insulin resistance (Lima et al. 2021). Studies have shown that excessive ROS production arises from nonmitochondrial and mitochondrial pathways. Mitochondrial dysfunction, in particular, contributes to intracellular calcium imbalances, which further reduces insulin sensitivity in patients with T2DM (Wang et al. 2017b). The inhibition of antioxidant enzymes by ROS and the depletion of nonenzymatic antioxidants in systemic tissues perpetuate chronic oxidative stress, intensifying the systemic complications associated with diabetes. Thus, overproduction of hyperglycemia-induced reactive oxygen species undermines the body's antioxidant defenses and triggers a detrimental cycle of hyperglycemia, oxidative stress, metabolic impairment, exacerbated insulin resistance, or reduced insulin secretion. Numerous molecular and signaling mechanisms have been related to the onset and advancement of osteosarcoma in periodontitis linked to diabetes mellitus (George et al. 2021; Su et al 2019).

### OS in periodontitis

Multiple experimental and research studies have established a significant correlation between periodontitis and OS (Wang et al. 2017a). Growing evidence indicates that heightened oxidative stress indicators and total oxidation levels are present in the blood, saliva, and GCF of periodontitis patients, hence reinforcing the association between periodontal tissue inflammation and oxidative stress (Wei et al. 2021). Periodontitis causes chronic, low-level inflammation of the periodontal tissues, resulting in heightened ROS generation and an impaired antioxidant defense mechanism. Nonsurgical periodontal therapy has been shown to reduce oxidative stress markers within periodontal tissues. Periodontal microbial infections activate the host immune response, leading to the recruitment of leukocytes, particularly polymorphonuclear neutrophils and macrophages, to the site of infection (Silva et al. 2018; Serhan and Levy 2018). Neutrophils interact with periodontal pathogens through pattern recognition receptors on their surface, such as TLR2 and TLR4, and subsequently eliminate the pathogens via phagocytosis, bactericidal activity, and the formation of NETs (Castanheira and Kubes 2019; DeLeo and Allen 2020; Wong and Wagner 2018). This process involves intense intracellular killing and a respiratory burst by neutrophils, resulting in the excessive release of ROS that assist in microbial elimination. However, the overproduction of ROS and the consequent oxidative stress can also be detrimental to host cells, functioning as a double-edged side (Ebersole et al. 2021; Fang et al. 2020). In line with the role of oxidative stress in diabetes, it can trigger the production of proinflammatory molecules and activate the NF-kB signaling pathway within periodontal tissue. ROS exerts antimicrobial effects by promoting nucleic acid damage, protein misfolding, lipid peroxidation, and stress in the endoplasmic reticulum or mitochondria. These processes, in turn, accelerate autophagy and apoptosis of damaged cells (Ebersole et al. 2021; Kuo et al. 2020; Portes et al. 2021). Furthermore, ROS production may indirectly facilitate the degradation of alveolar bone in periodontitis by acting as signaling molecules in the osteoclastogenesis of periodontal ligament stem cells (Graves et al. 2019; Han et al. 2018; Huang et al. 2020; Ohgi et al 2018; Jadson et al. 2010; Eid et al. 2010). Furthermore, excessive ROS production can trigger inflammatory processes and stimulate osteoclastogenesis, contributing to periodontal inflammation and the loss of alveolar bone as periodontitis progresses (Graves et al. 2018; Han et al. 2018; Polak et al. 2020a; Wu et al. 2019).

## OS in periodontitis associated with DM

Preexisting periodontal disease, together with the diverse pathological pathways associated with diabetes, such as elevated oxidative stress and increased secretion of proinflammatory mediators, may elucidate why diabetic patients endure more severe periodontal damage and exhibit greater susceptibility to periodontitis (Alshial et al. 2023; Ellakwa et al. 2024; Madkour et al. 2024; Mohamed et al. 2023; Safwat et al. 2024; Sanaeifar et al. 2024; Sayed et al. 2024; Soliman and Abdellatif 2023; Wadan et al. 2024a; Tolba et al. 2023; Wadan et al. 2024b). AGEs, a key pathological factor, plays a central role in linking diabetes with its complications. Studies have shown that in chronic periodontitis associated with diabetes, there is an accumulation of AGEs in the periodontium (Roy et al. 2019). The synergistic interaction between ROS and the AGE/RAGE pathway may contribute to substantial periodontal damage in individuals with diabetes (Altıngöz et al. 2021). Additionally, in the presence of AGEs, ROS can promote cellular autophagy by activating the ERK pathway and altering oxygen diffusion through cell membrane permeability and structure changes. These alterations further exacerbate oxidative stress within periodontal tissues. Research has also demonstrated that in diabetic conditions, AGEs accumulate in periodontal tissues and induce oxidative stress by inhibiting the Sirt1/Nrf2/HO-1 signaling axis via RAGE, which in turn promotes proinflammatory cytokines production such as IL-6 and IL-8 (Chen et al 2018). AGEs, in combination with TNF-α, can further amplify OS in human PDLSCs in vitro, resulting in more significant periodontal tissue damage (Roy et al. 2019; Thomas et al. 2014). Additionally, AGEs can hinder the osteogenic differentiation of PDLSC by inducing OS. Another study found that the excessive aggregation of ROS in periodontal tissues, induced by diabetes, creates a persistent oxidative environment, leading to telomere damage in PDLSCs, which ultimately impairs the repair and regenerative capacity of periodontal tissue (Fang et al. 2020; Guo et al. 2019).

### Antioxidants

A promising strategy for enhancing the defense against OS involves supplementing with antioxidants such as vitamins C and E, carotenoids (Black et al. 2020), lycopene (Black et al. 2020; Juiz et al. 2024; Malcangi et al. 2023), α-and-tocopherol, β-cryptoxanthin, NAC, polyphenolic compounds (Da Porto et al. 2021), such as flavonoids (Al-Ishaq et al. 2019), zeaxanthin (Black et al. 2020), and lutein. Although NAC alone has limited antioxidant effectiveness, its combination with glycine to form GlyNAC has been demonstrated to improve mitochondrial function synergistically, enhance insulin secretion, increase GPx synthesis (Black et al. 2020), and reduce OS (Sekhar 2022). Nrf2, a transcription factor associated with inflammation, is pivotal in mitigating oxidative stress damage and regulating antioxidant responses. Thus, targeting the Nrf2/HO-1 axis offers a potential therapeutic approach to alleviate periodontal damage caused by OS and mitigate the amplified inflammatory response in diabetic patients with periodontitis (Chen et al. 2018; Hsu et al. 2012; Mo et al. 2013; Vomhof-DeKrey and Picklo Sr 2012). Magnolol, derived from Magnolia officinalis, also improves diabetic complications (Kuo et al. 2020; Szu-Han et al. 2021).

## Epigenetic changes

Periodontal microorganisms and other factors, including genetic and environmental influences, epigenetic changes, immune dysregulation, inflammation, and metabolic disorders influence the host's susceptibility to periodontitis. Recent studies have emphasized that diabetes influences the initiation and advancement of periodontitis by modifying the epigenetics of periodontal tissue (Han et al 2011). Epigenetic changes and physical alterations in periodontal tissue may heighten the vulnerability to periodontal disease in individuals with diabetes (Li et al. 2021).

## Alveolar bone resorption and damage

Periodontitis in diabetic patients leads to more severe damage to periodontal tissues, particularly the alveolar bone, compared to periodontitis alone. The likely mechanisms contributing to the increased destruction of periodontal tissue in diabetes include reduced collagen synthesis, enhanced collagen degradation, RANKL-mediated osteoclastogenesis, and impaired bone regeneration (Barutta et al. 2022). RANKL primarily mediates alveolar bone resorption through its receptor RANK, while OPG is an antagonist to RANKL, inhibiting osteoclast formation and playing a key role in bone protection. The RANKL/OPG ratio influences bone metabolism, with this pathway being central to diabetic periodontal inflammation and tissue destruction (Barutta et al. 2022). Almeida et al. discovered that the levels of RANKL and the RANKL/OPG ratio in the GCF of periodontitis patients with poorly controlled diabetes were elevated compared to those in patients with well-controlled diabetes (Jadson et al. 2010). A separate study demonstrated that oral infection elevated RANKL expression in osteocytes, resulting in considerable bone loss and heightened osteoclast activity, with these effects intensified by diabetes (Wang et al. 2017a; 2017b; Wong and Wagner 2018; Zhang et al. 2023a, b, c). In diabetic transgenic mice devoid of RANKL in osteocytes, neither bone loss nor an increase in osteoclastogenesis was detected (Graves et al. 2018; Zhang et al. 2023a, b, c). In periodontitis-afflicted rats with T1DM, TNF-α stimulated heightened production of sclerostin and RANKL in alveolar osteocytes, hence exacerbating alveolar bone resorption and loss (Ateeq et al. 2022; Han et al. 2018; Polak Lior 2017; Jadson et al. 2010). Additionally, hyperglycemia associated with diabetes impairs the function of PDLSCs, negatively affecting osteogenic differentiation and hindering alveolar bone regeneration (Tang et al. 2022).

## Host Immunity

### Pathogenesis of periodontitis

The interaction between periodontal bacteria and the host immune response is essential for the onset and progression of periodontitis. The intensity of the host’s immune reaction to dysbiotic microbiota is a critical factor in determining the clinical outcomes of periodontitis and influencing the success of periodontal treatments (Bao et al. 2022; Lamont et al. 2018; Yamazaki et al. 2021). Excessive immune responses can intensify inflammation and accelerate tissue degradation in the periodontium (Madkour et al. 2024). Periodontal pathogenic bacteria are thoroughly recognized by pattern recognition receptors, such as TLR2, TLR4, TLR7, TLR8, and TLR9, on periodontal cells. This recognition triggers proinflammatory responses, activates neutrophils, and recruits macrophages and lymphocytes through the p38/MAPK and NF-κB signaling pathways (Bae et al. 2013; Cheng et al 2006; Cui et al 2009). Furthermore, PGE2 secreted by macrophages and OS instigated by neutrophils further stimulate the secretion of common proinflammatory cytokines including IL-6, TNF-α, IL-1β and along with MMPs such as MMP-1, MMP-2, MMP-8, MMP-9, and MMP-13 (Munhoz et al. 2007; Pasceri et al. 2000; Vaamonde-Garcia et al. 2017), which leads to the local degradation and disintegration of collagen fibers (Deng et al. 2021; Lima et al. 2021), loss of clinical periodontal attachment (Hiroshima et al. 2017; Toniolo et al. 2018), alveolar bone resorption, and contribute to the progression of periodontitis, amplifying the inflammatory response and tissue destruction (Acharya et al. 2016; Donath 2013; Fang et al. 2020; Longo et al. 2018; Loos Loos and Thomas 2020; Masi et al. 2018; Nicu and Loos 2016; Olsen et al. 2017; Patil et al. 2016). The inflammatory response initiated by the dysbiotic microbiota also encompasses IL-23 (Lamont et al 2018; Hajishengallis Triantafyllos 2021), produced by macrophages and dendritic cells in the connective tissue, which increases the proportional existence of Th17 lymphocytes and shifts the Th17/Treg balance toward excessive proinflammation by completely turning into Th17 cells. IL-17 is crucial for periodontal destruction as it mediates collagen degradation via neutrophils, promotes alveolar bone loss through the RANK/RANKL pathway, and further contributes to periodontitis (Ateeq et al. 2022; Graves et al. 2019; Sereti 2020). Host genotype may also play a significant role in periodontal susceptibility (Renvert and Quirynen 2015)., while comorbidities like diabetes and environmental factors, including susceptibility to smoking, are also critical contributors.

### DM is involved in the periodontal immune response

DM can affect both the innate and adaptive immune systems in the normal periodontium, exacerbating the course of periodontitis. Neutrophils, an essential element of innate immunity, serve as the primary defense against periodontal bacteria (Castanheira and Kubes 2019). Maintaining neutrophil homeostasis is essential for balancing protection and tissue damage; any imbalance can destroy periodontal tissue. Previous studies indicated that while the number of neutrophils is increased in periodontitis patients with T2DM, their functions, such as chemotaxis, phagocytosis, and bactericidal activity, are impaired (Manosudprasit et al. 2017). This deficiency leads to impaired intracellular killing and a reduced respiratory burst, exacerbating the severity of periodontitis in DM patients. Dysfunctional neutrophils may further aggravate periodontal tissue damage by releasing inflammatory mediators and enzymes that degrade tissue (Munhoz et al. 2007). In patients with poorly controlled diabetes, neutrophils can be prematurely activated, increasing periodontal damage by enhancing PKC activity (Wang et al. 2011). These impaired neutrophils amplify tissue damage by producing excessive superoxide, proinflammatory cytokines, and chemokines and by increasing neutrophil numbers in the periodontal tissue (Castanheira and Kubes 2019; Nicu and Loos 2016; Silva et al. 2018; Sima et al. 2016). Additionally, neutrophils in diabetic patients overexpress PAD4, an enzyme involved in chromatin decondensation. This disruption weakens the periodontal defense response, exacerbates inflammation, and facilitates the formation of NETs. These NETs and fibrin contribute to the host's defense against pathogens. Furthermore, the levels of calprotectin (S100A8/A9), a critical cytoplasmic protein in neutrophils, are significantly elevated in periodontitis patients with T2DM compared to individuals with chronic periodontitis or healthy controls (Gao et al. 2020).

**Fig. 2:**
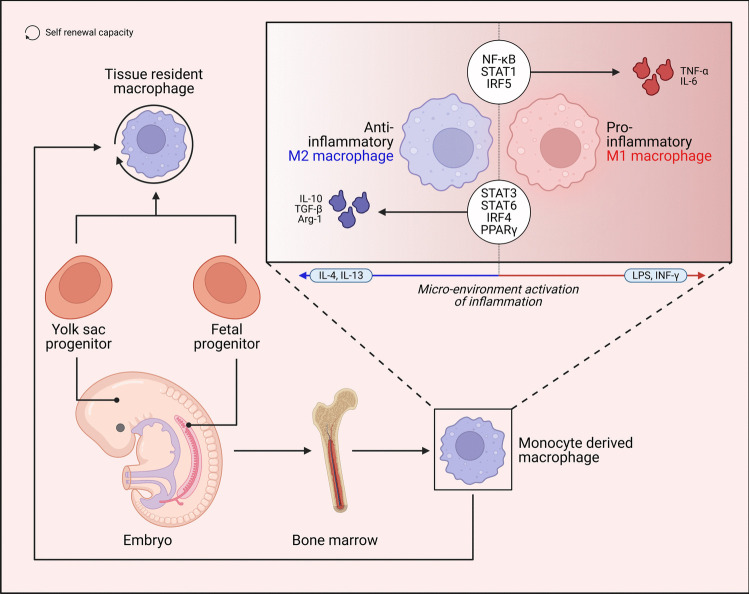
Origin and plasticity of macrophages. Tissue-resident macrophages originate from monocytes generated from bone marrow, yolk sacs, and the fetal liver. These cells differentiate into two primary extreme states, M1 and M2, exhibiting pro-inflammatory and anti-inflammatory functions, respectively

Macrophages, another crucial type of immune cell, play a significant role in diabetes-associated periodontitis. Diabetes can promote the polarization of macrophages toward the proinflammatory M1 phenotype, which further amplifies the inflammatory response and accelerates tissue damage in periodontitis (Graves et al. 2019), heightening the vulnerability and intensity of the periodontal disease, concomitantly with a decrease in the quantity of anti-inflammatory M2 macrophages (Yin et al. 2022). M1 macrophages are involved in inflammation, whereas M2 macrophages help resolve inflammation (Fig. 2) (Russo 2021). Diabetes-induced systemic metabolic alterations, such as hyperglycemia and insulin resistance, modify macrophage polarization and function, augment myelopoiesis, and elevate the release of monocytes that develop into macrophages (Flynn et al. 2020). Studies have demonstrated that in patients with diabetic periodontitis, macrophages exposed to elevated glucose levels exhibit reduced secretion of SIRT6, disrupting the SIRT6-miR-216/217 axis. This disruption contributes to neutrophil apoptosis, impaired efferocytosis, and an increased formation of neutrophil extracellular traps, all of which exacerbate the inflammatory response in periodontitis (Li et al. 2023). Neutrophils eliminate periodontal pathogens through phagocytosis or the release of NETs, which triggers apoptosis and efferocytosis by macrophages to prevent additional tissue damage. However, in chronic inflammation, commonly observed in diabetes, the prolonged presence of neutrophils and macrophages exacerbates periodontal inflammation (Li et al. 2023).

While neutrophils and macrophages are essential to the host immune response, other myeloid cells, including T cells, significantly contribute to periodontitis linked to DM. The inflammatory environment, maintained by innate immune cells such as neutrophils and macrophages, along with the associated inflammatory mediators, can be modulated by adaptive immunity. In individuals with diabetes, adaptive immune responses drive the differentiation of CD4 + T cells into Th17 inflammatory cells, which disrupts the balance of the Th17/Treg axis and increases the proportion of Th17 cells (Tarannum Mohamed 2017). IL-17 alters the pathogenicity of the periodontal microbiota; this leads to increased inflammation and more significant periodontal bone loss. The elevated Th17/Treg ratio further intensifies the progression of periodontitis in diabetes (Amaral et al. 2018).

## Host-response modulation

Host response modulation therapy seeks to restore balance between proinflammatory agents and anti-inflammatory mediators, prevent the progression of periodontitis, and promote the regeneration of periodontal tissue damage (Golub Hsi-Ming 2019). The primary substances utilized in host response modulation therapy include NSAIDs (Petit et al. 2019), anti-cytokine therapy (Curtis et al. 2020), sub-antimicrobial doses of SDD (Emingil et al. 2019), and specialized pro-resolving mediators (SPM). SPMs include resolvins (Alshibani 2022; Serhan and Levy 2018), maresins (Gireddy et al 2022), lipoxins (Osorio 2018; Tarannum Mohamed 2017; Van Dyke 2017), probiotics (Corbella et al 2021; Han et al 2011). Other antioxidant substances include resveratrol (Alshial et al. 2023), melatonin (Madkour et al. 2024), and curcumin (Solomon et al. 2022; Van Dyke 2020) are discussed in (Table 1).

**Table 1:** The primary types of substances employed in host response modulation for patients with diabetes-related periodontitis include

Drug/Substance	Reference/ Study	Mode of Action	Clinical/Preclinical Trials	Periodontitis Stage	Molecular/Inflammatory Pathways
Doxycycline	(Matta Reddy et al. 2022)	MMP inhibitor; Reduces collagen breakdown in connective tissues	Clinical: FDA-approved sub-antimicrobial doses tested for diabetic patients	Moderate to severe	Down-regulates MMPs reduces TNF-α and IL-1β levels
Omega-3 fatty acids	(Kruse et al. 2020)	Anti-inflammatory; Promotes resolution of inflammation	Clinical: Omega-3 + aspirin tested in type 2 diabetes and periodontitis	Mild to moderate	Decreases IL-6, TNF-α, C-reactive protein (CRP), downregulates NF-κB signaling
Curcumin	(Zhang et al. 2022)	Anti-inflammatory, antioxidant, reduces oxidative stress	Preclinical: Animal models; some clinical trials ongoing in diabetic patients	Moderate to severe	Down-regulates NF-κB, TNF-α, IL-6, inhibits ROS production
Resveratrol	(Andrade et al. 2019)	Anti-inflammatory, antioxidant, and SIRT1 activator	Preclinical: Some clinical trials underway in periodontal therapy	Moderate	Down-regulates IL-1β, IL-6, TNF-α; increases SIRT1 activation, reduces NF-κB
Metformin	(Najeeb et al. 2018)	Anti-inflammatory; increases insulin sensitivity	Clinical: Studies in diabetic patients with periodontitis	Mild to moderate	Decreases IL-1β, TNF-α, IL-6, and upregulates AMPK signaling pathways
Aspirin (Low-dose)	(Castro Dos Santos et al. 2020)	Anti-inflammatory reduces platelet aggregation, promotes resolution of inflammation	Clinical: Tested with omega-3 in diabetic patients	Mild to moderate	Decreases CRP, TNF-α, and IL-6; promotes lipid mediators of inflammation resolution (e.g., resolvins)
Probiotics (e.g., Lactobacillus)	(Invernici et al. 2018)	Balances oral microbiota, anti-inflammatory effects	Clinical: Tested in diabetic patients with mild periodontitis	Early to moderate	Down-regulates IL-6, TNF-α; promotes regulatory T cells (Tregs)
Vitamin D	(Lu 2023)	Anti-inflammatory; enhances bone metabolism and immune response	Clinical: Ongoing studies in diabetic periodontitis patients	Moderate to severe	Decreases pro-inflammatory cytokines (IL-1β, IL-6, TNF-α); promotes bone health and calcium metabolism
Melatonin	(Cutando et al. 2014)	Antioxidant modulates immune response, reduces oxidative stress	Preclinical: Animal models and some clinical trials in diabetic patients	Moderate to severe	Reduces ROS, down-regulates pro-inflammatory cytokines like IL-1β, IL-6, and TNF-α

## The involvement of microbiome factors

Microbial dysbiosis is crucial in the pathogenesis of periodontitis, as the transition from beneficial symbiotic microbial communities to pathogenic bacteria within subgingival plaque biofilms is the main driver of the disease. These microbial communities adhere to the root surfaces of the teeth, where they are more effectively shielded from shear forces and ambient oxygen than those found in supragingival areas (Chen et al. 2020; Lamont et al. 2018; Qin et al. 2022). It is well-established that periodontal microbial biofilms can induce host inflammation, resulting in periodontal damage and tooth loss. Although earlier studies were unclear about the influence of DM on the composition of the periodontal microbiota (Neto et al. 2022; Qin et al. 2022; Yu et al. 2022). Some researchers have argued that insufficient evidence shows that DM significantly affects the periodontal microbiota. Furthermore, they found that the level of glycemic control in diabetic patients did not influence the composition of the subgingival bacterial biofilm (Polak Lior 2017). Recent next-generation sequencing technologies (NGST) advancements have provided more precise insights. These results indicate that diabetes mellitus can modify the makeup and biodiversity of the subgingival microbiome. Periodontal disease and varying glycemic levels dramatically affect microbiota composition. Individuals with diabetes exhibited elevated levels of Actinobacteria and Fusobacterium, with Actinobacteria raising the probability of diabetes by 10% and Fusobacterium by 14%, although Proteobacteria were less prevalent (Matsha et al. 2020). Other research highlighted significant differences in the salivary microbiomes between nondiabetic individuals and those with a history of diabetes (Neto et al. 2022). Diabetes and pre-diabetes were linked to diminished biological and phylogenetic diversity in the subgingival microbiota relative to normoglycemic people (Saeb Khalid 2019). Moreover, diabetes amplifies the pathogenic potential of the oral microbiota by increasing the production of IL-17. Administration of IL-17 antibodies has been demonstrated to diminish the pathogenicity of subgingival microbiota, reduce neutrophil recruitment, and lower levels of pro-inflammatory factors such as IL-6 and RANKL. This, in turn, reduces alveolar bone resorption and mitochondrial dysfunction (Curtis et al. 2020).

## Inflammatory mechanisms

Microbial dysbiosis not only directly impacts periodontal tissues but also has a pivotal role in intensifying the host's inflammatory response through the formation of integrating adhesive biofilms on tooth surfaces, which further drive the progression of periodontitis in susceptible individuals (Curtis et al. 2020). The buildup of dental plaque stimulates inflammation in the periodontal tissues, creating an environment conducive to the growth of gram-negative bacteria. This unchecked inflammatory response and immune reaction play a significant role in tissue damage (Curtis Patricia and Dyke 2017). Inflammation is a defensive response of the periodontal support tissues to bacterial threats, designed to restore homeostasis by removing harmful stimuli, such as infections (Alqerban 2020). However, chronic inflammation, frequently exacerbated by conditions like DM, leads to excessive tissue damage (Kornman et al. 2020).

### Inflammatory cytokines

There is substantial evidence indicating that, compared to individuals with isolated periodontitis, patients with both diabetes and periodontitis exhibit higher levels of proinflammatory cytokines such as IL-6 and IL-1β, with a direct correlation between glycemic control and the concentration of these cytokines (Taylor et al. 2013). Conversely, reduced secretion of anti-inflammatory cytokines like TGF-β, IL-4, and IL-10 (Table 2) may negatively contribute to exacerbating periodontal inflammation in diabetic patients (Acharya et al. 2016).

**Table 2:** Shows all the available anti-inflammatory substances and molecules that target treating mitochondrial dysfunction associated with diabetes and periodontitis

Anti-inflammatory mediator			Type	Mode of Action	Mitochondrial Pathways Supported	Reference
1.	IL-10	Cytokine		Anti-inflammatory signaling	Enhances mitochondrial biogenesis	(214,215)
2.	IL-4			Modulates macrophage polarization	Maintains mitochondrial membrane integrity	(Bailey et al. 2004; Black et al. 2020)
3.	TGF-β			Suppresses immune response	Protects mitochondrial DNA	(Casalena et al. 2012; Guak et al. 2022; Zhu et al. 2010)
4.	Resveratrol	Polyphenol		Activates SIRT1	Promotes mitochondrial biogenesis	(Zhou et al. 2021)
5.	Curcumin			NF-κB pathway inhibitor	Reduces mitochondrial oxidative stress	(Hou et al. 2024)
6.	Omega-3 Fatty Acids	Fatty acid		Reduces inflammation	Protects mitochondrial membrane	(Afshordel et al. 2015; Lepretti et al. 2018; Tachtsis et al. 2020)
7.	Melatonin	Hormone		Free radical scavenger	Protects mitochondrial integrity	(Das et al. 2017)
8.	Vitamin D	Vitamin		Modulates immune response	Supports mitochondrial function	(Elenkova et al. 2018; Lu 2023; Reddy et al. 2022)
9.	Vitamin E	Antioxidant		Reduces oxidative damage	Prevents lipid peroxidation	(Chow et al. 1999)
10.	Nrf2 Activators	Protein activator		Regulates antioxidant response	Increases mitochondrial resilience	(Holmström et al. 2016; Vomhof-DeKrey and Picklo Sr 2012)
11.	Catalase	Enzyme		H2O2 decomposition	Enhances mitochondrial detoxification	(Salvi et al. 2007)
12.	SOD			Converts superoxide to H2O2	Reduces mitochondrial oxidative stress	(Vorotnikova et al. 2010)
13.	GPx (Glutathione Peroxidase)			Neutralizes H2O2	Protects mitochondrial DNA	(Marí et al. 2009)
14.	PGC-1α	Protein		Regulates mitochondrial biogenesis	Promotes mitochondrial function	(Koh and Kim 2021)
15.	SIRT3	Enzyme		Deacetylates proteins	Supports mitochondrial homeostasis	(Pillai et al. 2016)
16.	Coenzyme Q10	Cofactor		Antioxidants in the electron transport chain	Supports mitochondrial ATP synthesis	(Mantle et al. 2024)
17.	Taurine	Amino acid		Reduces inflammation and ROS	Preserves mitochondrial membrane	(Jong et al. 2021)
18.	Glutathione	Antioxidant		Neutralizes ROS	Protects mitochondrial DNA	(Ceballos-Picot et al 1996)
19.	N-acetylcysteine (NAC)			Increases glutathione	Protects mitochondrial membrane	(Sekhar 2022)
20.	Fisetin	Flavonoid		Anti-inflammatory and antioxidant	Protects mitochondrial function	(Ay 2023)
21.	PPAR-γ Agonists	Nuclear receptor		Reduces inflammation	Protects mitochondrial function	(Corona and Duchen 2016)
22.	Aspirin	Anti-inflammatory		COX inhibition	Protects mitochondrial DNA	(Suzuki et al. 2010)
23..	5-ASA			Scavenges free radicals	Protects mitochondrial membrane	(Castro Dos Santos et al. 2020)
34.	Quercetin	Flavonoid		Reduces pro-inflammatory cytokines	Supports mitochondrial function	(Houghton et al. 2018; Wei et al. 2021)
35.	EGCG (Green Tea Extract)			Reduces oxidative damage	Protects mitochondrial membrane	(James et al. 2023)
36.	Astaxanthin	Carotenoid		Antioxidation	Supports mitochondrial health	(Nishida et al. 2021)
37.	Lycopene			Neutralizes free radicals	Protects mitochondrial DNA	(Prakash and Kumar 2013)
38.	Zinc	Mineral		Antioxidant	Supports mitochondrial function	(Liu et al 2021; Patr et al. 2022)
39.	Selenium				Protects mitochondrial DNA	(Mehta et al. 2012; Zhang et al. 2021)
30	Apigenin	Flavonoid		Anti-inflammatory	Supports mitochondrial function	(Naponelli et al. 2024)
41	Bilirubin	Pigment		Free radical scavenger	Protects mitochondrial membrane	(Naponelli et al. 2024)
42.	Rutin	Flavonoid		Antioxidant	Supports mitochondrial function	(Gong et al. 2010)
43.	Omega-6 Fatty Acids	Fatty acid		Reduces inflammation	Maintains mitochondrial homeostasis	(Lepretti et al. 2018)
44.	Glucosamine	Amino acid		Reduces cytokine expression	Protects mitochondrial function	(Chiu et al. 2019)
45.	Alpha-lipoic acid	Antioxidant		Neutralizes ROS	Protects mitochondrial DNA	(Dos Santos et al. 2019)
46.	Berberine	Alkaloid		Activates AMPK	Protects mitochondrial membrane	(Chen et al. 2008; Jiang et al. 2015; Kim et al 2007)
47.	Boswellic Acid	Triterpene		Inhibits pro-inflammatory cytokines	Maintains mitochondrial integrity	(Siddiqui et al. 2021)
48.	Aloe Vera	Plant extract		Anti-inflammatory	Protects mitochondrial membrane	(Zhang et al. 2023a)
49.	Zinc Oxide	Mineral compound		Antioxidant enzyme cofactor	Supports mitochondrial ATP production	(Patr et al. 2022)
50.	Vitamin C	Vitamin		Neutralizes free radicals	Supports mitochondrial function	(Toraman et al. 2020)
51.	Boswellia Extract	Plant extract		Inhibits pro-inflammatory mediators		(Gunasekaran et al. 2021)
52.	Aloe Vera Gel			Reduces oxidative stress	Protects mitochondrial membrane health	(Zhang et al. 2023b)
53.	Apocynin	Antioxidant		NADPH oxidase inhibitor		(Zhang et al. 2023c)
54.	Tocotrienols	Vitamin E variant		Antioxidant	Supports mitochondrial function	(Nowak et al. 2012)
55.	Hesperidin	Flavonoid		Reduces pro-inflammatory cytokines	Supports mitochondrial detoxification	(Nowak et al. 2012)

In addition to cytokines, other pro-inflammatory mediators, such as MMPs and chemokines, are implicated in diabetes (Table 3). Studies have MMP-14 expression is significantly elevated in the periodontal tissue of diabetic patients with decreased glycemic control (Samah et al. 2023). On the contrary, a reduction in pro-resolution mediators such as resolvins (Alshibani 2022; Serhan and Levy 2018), protectins (Gireddy et al 2022; Serhan and Levy 2018), maresins (Gireddy et al 2022), and lipoxins (Chiang and Serhan 2020) may contribute to increased periodontal inflammation. These mediators belong to three distinct biosynthetic families with potent effects. During the inflammatory process, proinflammatory factors signal the production of pro-resolution mediators such as resolvins, activating the innate immune system to eliminate pathogen stimuli (Alshibani 2022; Chiang and Serhan 2020; Lee et al. , 2013; Serhan and Levy 2018; Van Dyke 2017). Further research has substantiated that concentrations of the proinflammatory peptide substance-P are heightened in the GCF and serum of diabetic patients exhibiting inadequate glycemic control, potentially exacerbating the advancement of periodontitis and resulting in increased periodontal destruction (Akram et al. 2019; Chen et al. 2019; Gao et al. 2020; Govindaraj et al. 2021; Hiroshima et al 2012; Radović et al. 2018; Sereti 2020).

**Table 3:** Shows proinflammatory mediators related to periodontitis associated with DM that cause the overproduction of mitochondrial ROS leading to mitochondrial dysfunction

Proinflammatory mediator		Type	Mode of Action	Involvement in ROS Production	Mitochondrial Pathways Affected	References
1.	TNF-α	Cytokines	Inflammatory signaling via NF-κB	Promotes ROS production	Mitochondrial membrane permeability, ATP production	(Amasheh et al. 2010; Xue et al. 2005)
2.	IL-1β		Induces inflammatory genes	Increases ROS via NADPH oxidase	Affects mitochondrial respiratory complexes	(Deng et al. 2024; Portes et al. 2021)
3.	IL-6		Triggers acute phase response	Increases mitochondrial superoxide	Modulates mitochondrial bioenergetics	(Portes et al. 2021; Yin et al. 2022)
4.	IL-17		Amplifies inflammatory cell recruitment	Enhances ROS generation	Mitochondrial ETC	(Ding et al. 2022; Portes et al. 2021; Qin et al., 2022)
5.	INF-γ		Activates macrophages	Increases ROS, Nitric oxide	Disrupts mtDNA	
6.	MMP-9	Enzyme	Degrades ECM	Indirect ROS generation through tissue damage	Alters mitochondrial structural integrity	(Deng et al. 2021; Emingil et al. 2019)
7.	CRP	Acute phase protein	Enhances leukocyte adhesion	Increases ROS and inflammatory signaling	Affects mitochondrial dynamics	(Khalili et al. 2021; Polak Lior 2017)
8.	NF-κB	Transcription factor	Induces pro-inflammatory cytokines	Upregulates mitochondrial ROS generation	Impacts mtDNA and function	(Mann et al 2017; Xie et al. 2013)
9.	PGE2	Lipid mediator	Inflammatory prostaglandin	Increases ROS production	Affects mitochondrial membrane potential	(Vaamonde-Garcia et al. 2017)
10.	ROS	Reactive species	Activates inflammasome	Primary cause of mitochondrial dysfunction	Causes mtDNA damage, impacts ATP generation	(Li and Shah 2003a, 2003b, 2003c)
11.	LPS	Bacterial endotoxin	TLR4 activation, inflammatory response	Triggers ROS and NO release	Affects mitochondrial oxidative phosphorylation (OXPHOS)	(Ke et al 2014; Remppis et al. 2010)
12.	NO	Reactive species	Free radical, reacts with superoxide	Forms peroxynitrite, damages mitochondria	Alters mitochondrial DNA reduces bioenergetics	(Lee et al. , 2013; Perreault André, 2001)
13.	IL-18	Cytokine	Activates inflammatory cascade	Enhances ROS, amplifies inflammation	Disrupts mitochondrial structure and function	(García-Hernández al et al. 2018)
14.	MCP-1	Chemokine	Monocyte recruitment	Indirectly enhances ROS production through macrophage activity	Alters mitochondrial integrity	(Ding et al. 2022; Wang 2013)
15.	IL-12	Cytokine	Induces T-cell proliferation	Contributes to ROS production	Affects mitochondrial respiration	(Kashiwagi et al. 2016)
16.	IL-23		Promotes Th17 cell response	Enhances ROS via Th17 activity	Disrupts mitochondrial membrane potential	(Kashiwagi et al. 2016)
17.	TGF-β		Immunosuppressive, fibrosis promoter	Modulates oxidative stress	Inhibits mitochondrial biogenesis	(Liu et al., 2022a)
18.	Fasl	Apoptotic ligand	Induces apoptosis	Increases ROS leading to cell death	Affects mitochondrial permeability	(Manosudprasit et al. 2017)
19.	HMGB1	Nuclear protein	DAMP, initiates inflammation	Increases ROS and mitochondrial damage	Affects mitochondrial ATP production	(Tang et al. 2011)
20.	IL-8	Chemokine	Neutrophil recruitment	Enhances ROS via neutrophils	Alters mitochondrial membrane integrity	(Kashiwagi et al. 2016)
20.	COX-2	Enzyme	Produces pro-inflammatory prostaglandins	Induces ROS production	Impairs mitochondrial bioenergetics	(DuBois et al. 1998; Wei et al. 2021)
22.	ICAM-1	Adhesion molecule	Promotes leukocyte adhesion	Induces ROS through cell adhesion	Alters mitochondrial bioenergetics and function	(Nonaka et al. 2017; Pasceri et al. 2000; Tang et al. 2015)
23.	S100A8/A9	Protein	Calcium-binding proteins, inflammatory	Enhances ROS production in neutrophils	Disrupts mitochondrial membrane potential	(Flynn et al. 2020)
24.	TREM-1	Receptor	Amplifies TLR signaling	Enhances ROS and inflammatory cytokine release	Disrupts mitochondrial dynamics	(Zhong et al. 2023)
25.	GM-CSF	Cytokine	Activates granulocytes and macrophages	Induces ROS and pro-inflammatory signaling	Affects mitochondrial ATP generation	(Wessendarp et al. 2022)
26.	CD40L	Ligand	Co-stimulation enhances inflammation	Increases ROS via NF-κB	Impacts mitochondrial membrane integrity	(Chen et al. 2008)
27	IL-15	Cytokine	T-cell activation and proliferation	Induces mitochondrial ROS production	Affects mitochondrial energy generation	(Barra et al. 2014)
28	ICAM-3	Adhesion molecule	Leukocyte adhesion	Enhances ROS via cell adhesion	Affects mitochondrial dynamics	(Sapoznikov et al. 2023)
29	NLRP3	Inflammasome	Activates caspase-1, IL-1β release	ROS is a priming factor	Alters mitochondrial membrane potential	(García-Hernández al et al. 2018; Kuo et al. 2020)
30	TLR2	Receptor	Recognizes bacterial components	Induces ROS through inflammatory pathways	Affects mitochondrial bioenergetics	(Ohgi et al 2018)
31.	TLR4		Recognizes bacterial LPS	Induces ROS and NO production	Alter mitochondrial respiratory function	(Wang 2013)
32.	VEGF	Growth factor	Promotes angiogenesis	Increases ROS during inflammation	Alters mitochondrial bioenergetics	(Wang 2013)
33.	IL-10	Anti-inflammatory	Regulates immune response	Indirect ROS modulation	Balances mitochondrial bioenergetics	(Cherry and Piantadosi 2015)
34.	IL-33	Cytokine	Alarmin, amplifies the Th2 response	Increases ROS generation	Alters mitochondrial ATP production	(Xu et al. 2019)
35.	MMP-2	Enzyme	Degrades matrix proteins	Indirectly promotes ROS	Alters mitochondrial structure	(Samah et al. 2023)
36.	INF-α	Cytokine	Antiviral and immunoregulatory	Increases ROS in response to pathogens	Affects mitochondrial membrane potential	(Yanase et al. 2000)
37.	CXCL8	Chemokine	Neutrophil chemotaxis	Enhances ROS generation	Affects mitochondrial dynamics	(Yuan et al. 2024)
38.	BAFF	Cytokine	Promotes B-cell survival	Increases ROS through immune activation	Affects mitochondrial integrity	(Lempicki et al.^ 2022^)
39.	TIMP-1ss	Enzyme inhibitor	Inhibits MMPs	ROS modulation through MMP inhibition	Alters mitochondrial membrane integrity	(Ashutosh et al. 2012)
40.	PTX3	Protein	Acute phase protein, opsonin	Induces ROS production in macrophages	Alters mitochondrial ATP synthesis	(Carrizzo et al. 2019)
41.	HIF-1α	Transcription factor	Responds to hypoxia, induces VEGF	Affects mitochondrial metabolism and respiration.	HIF-1α mediates adaptation to hypoxic conditions by reducing mitochondrial activity	(Huang et al. 2020)

### The Involvement of Adipokines

There are various types of adipokines, including adiponectin (Wang et al. 2021; Wu et al. 2019) which is linked to metabolic-related function, resistin, and leptin (Al-Suhaimi Adeeb 2013) associated with endocrine function (Akram et al. 2016; Hiroshima et al 2012), complement factors related to response and interaction with immunity (Black et al. 2010), and angiotensinogen, which is involved in cardiovascular function (Roganović, 2021). Previous studies indicated that some adipokines contribute to the onset and progression of periodontal disease and diabetes by increasing mitochondrial ROS production (Wadan et al., 2024; Soliman and Abdellatif 2023; Wadan and Liaquat 2024). As a pro-inflammatory factor, resistin stimulates the release of IL-12 and TNF-α, activates NF-kB, and enhances the secretion of MCP-1 and IL-6 (Akram et al. 2016). Two studies found similar levels of resistin in the GCF and serum of periodontitis patients with and without diabetes (Hiroshima et al 2012). Both T2DM and periodontitis are characterized by decreased adiponectin levels and elevated leptin levels. Adipokines such as adiponectin, leptin, visfatin, and chemerin play pivotal roles in the pathogenesis and complications of T2DM. The APR activates endogenous adiponectin receptors, exerting osteoanabolic effects by significantly reducing osteoclast activity and preventing alveolar bone loss. Moreover, APR enhances the osteogenic differentiation of stem cells, downregulates SDF-1, which facilitates stem cell migration, and promotes alveolar bone repair and regeneration (Wu et al. 2019). Adiponectin and its agonists may offer promising therapeutic targets for treating diabetes-related periodontitis, with broad potential mechanisms and clinical applications (Wang et al., 2021).

## MicroRNAs

MicroRNAs (miRNAs) are small, non-coding RNA molecules that play a crucial role in regulating gene expression. They have been implicated in the development and progression of various diseases, including diabetes mellitus and periodontitis (Table 4), primarily through mechanisms associated with mitochondrial dysfunction (Amaral et al. 2018; Hashimoto Tomoaki 2016; Simarjit et al. 2017; Pu et al. 2018). Dysregulation of miRNAs, such as miR-146a, Mir-155, and miR-203 (Al-Rawi et al. 2020; Radović et al. 2018; Roganović, 2021), has been linked to chronic inflammatory diseases, influencing immune responses and inflammation critical to both conditions. MiR-146a regulates TLR signaling pathways and is associated with enhanced inflammatory responses to periodontal pathogens. Similarly, miR-21 inhibits pro-inflammatory cytokine production (Amaral et al. 2018), while the miR-200 family influences TLR-mediated signaling and may affect inflammatory responses in periodontal tissues (Matsui et al. 2018). Recent studies have identified specific salivary miRNAs as potential biomarkers for predicting periodontal disease severity in diabetic patients (Al-Rawi et al. 2020). Additionally, bioinformatics analyses have revealed that dysregulated miRNAs are involved in pathways related to inflammation and oxidative stress.

**Table 4:** Key miRNAs implicated in the reduction of mitochondrial ROS production, improvement of mitochondrial function, and potential therapeutic impact on periodontitis-associated DM

MiRNAs		Target Molecule in the Pathway	References
1.	miR-146a	NF-κB, TRAF6	(Roganović, 2021)
2.	miR-21	PTEN, NF-κB	(Wu et al. 2017b)
3.	miR-155	SOCS1, IKKε	(Mahesh Roopa 2019; Mikamori et al. 2017)
4.	miR-30a	Beclin-1, Drp1	(Li et al. 2010)
5.	miR-124	STAT3, IL-6	(Liang et al. 2023)
6.	miR-223	IL-1β, NLRP3	(Brook et al. 2019; Jiao et al. 2021)
7.	miR-34a	SIRT1, p53	(Payne et al. 2023)
8.	miR-126	VEGF, ICAM-1	(Accardi et al. 2022; Wu et al. 2017a)
9.	miR-15a/16	BCL2, MCL1	(Wen et al. 2022)
10.	miR-181b	NF-κB	(Indrieri et al. 2019)
11.	miR-125b	TRAF6, TNF-α	
12.	miR-210	HIF-1α, ISCU	(Song et al. 2022)
13.	miR-27a	PPARγ, UCP2	(Barisciano et al. 2021)
14.	miR-199a	HIF-1α, mTOR	(Yan et al. 2021)
15.	miR-145	TLR4, Smad3	(Han et al. 2021)
16.	miR-126-3p	VCAM-1, ROS pathways	
17.	miR-98	TNF-α, NF-κB	(Luan et al. 2018)
18.	miR-125a	TGF-β, Wnt signaling	(Pan et al. 2018)
19.	miR-29b	IGF-1R, mTOR	
20.	miR-99a	IGF-1R, mTOR	(Si et al. 2016)
20.	miR-19b	PTEN, BCL-2	(Saini et al. 2018)
22.	miR-218	RAGE, NF-κB	(Rita et al. 2020)
23.	miR-223-3p	IL-1β, TLR4	
24.	miR-142-3p	SOD2, ROS pathways	
25.	miR-18a	Smad3, TGF-β	
26.	miR-146b	TRAF6, IRAK1	
27	miR-106a	PGC-1α, NRF1	(Zhang et al. 2013)
28	miR-30d	Drp1, BAX	
29	miR-214	BCL2L11, p53	(Bai et al. 2019)
30	miR-379	PGC-1β, NRF2	(Sanson et al. 2020)
31.	miR-497	Bcl-2, GLUT1	(Yang et al. 2016)
32.	miR-133a	SIRT1, PGC-1α	(Liu et al. 2011)
33.	miR-375	JAK2, NF-κB	(Eid et al. 2010)
34.	miR-92a	VEGF, ICAM-1	(Li et al. 2019)
35.	miR-101	COX-2, MMP-9	(Liu et al. 2022b)
36.	miR-27b	PPARγ, ROS pathways	(D’Onofrio et al. 2023)
37.	miR-150	c-Met, VEGF	(Cron et al. 2019)
38.	miR-106b	p21, ROS pathways	(Zhang et al. 2013)
39.	miR-423-5p	BCL2, BAX	(Zhu and Lu 2019)

Each miRNA is listed alongside targeted molecules or pathways. The miRNAs listed here regulate various pathways—such as NF-κB, TGF-β, and PTEN—that influence inflammation, apoptosis, mitochondrial dynamics, and oxidative stress responses. By modulating these pathways, these miRNAs contribute to overall cellular and mitochondrial health, offering the potential to mitigate mitochondrial dysfunction and inflammation in periodontitis and DM.

## Alterations of mitochondrial quality control (MQC)

**Fig. 3:**
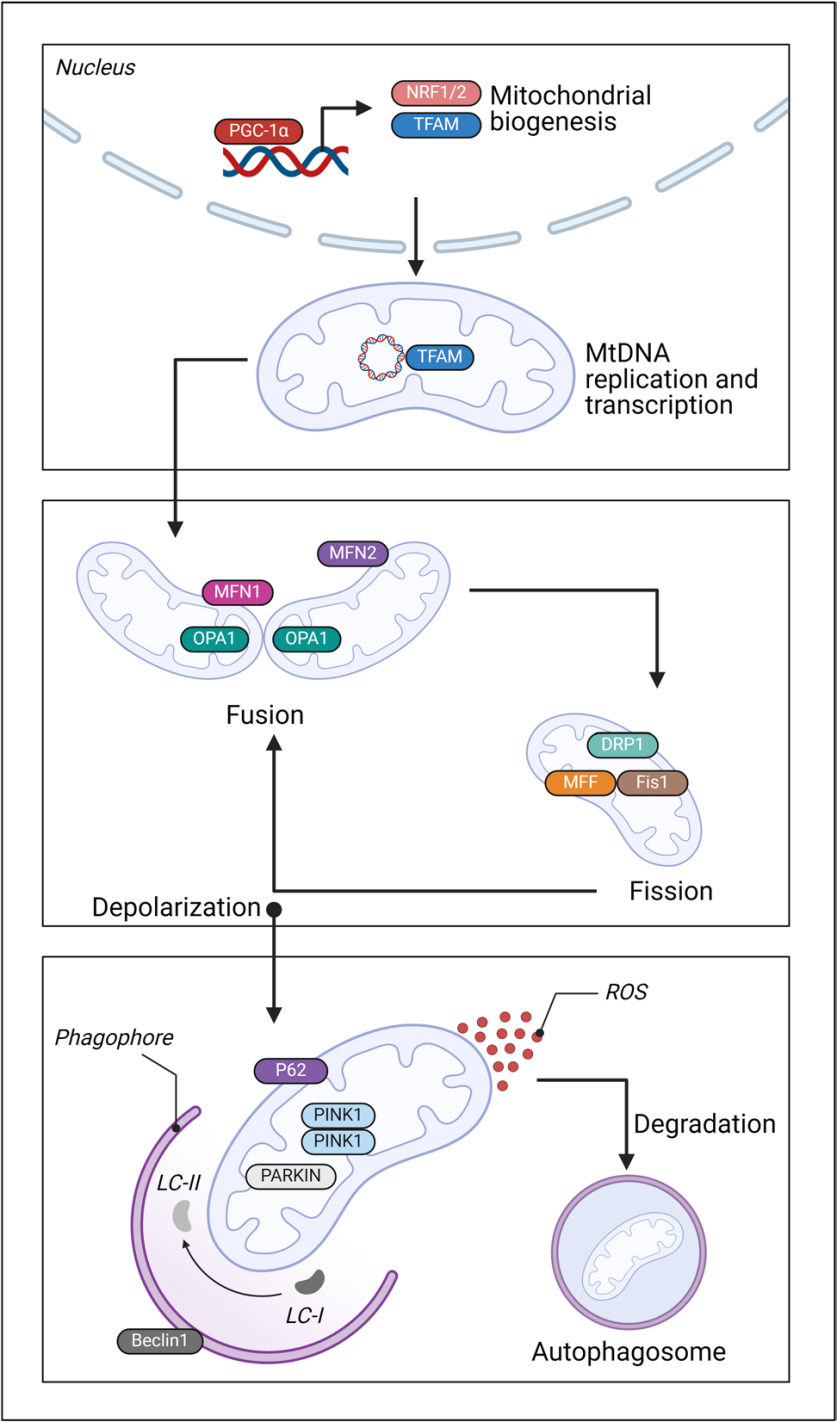
MQC involves several interconnected processes. Mitochondrial biogenesis is primarily regulated by PGC-1α, which stimulates the expression of NRF1, NRF2, and TFAM in the nucleus. The mitochondrial life cycle includes fusion and fission, critical for maintaining mitochondrial integrity. Fusion promotes the formation of elongated mitochondrial networks and is mediated by MFN1, MFN2, and OPA1. In contrast, fission, regulated by DRP1, FIS1, and MFF, leads to fragmented mitochondria and enables the isolation of damaged components. Throughout their lifespan and under pathological conditions such as CVDs, mitochondria accumulate oxidative damage. Fission allows the segregation of damaged mitochondria, which are subsequently eliminated via mitophagy. This process begins with the accumulation of PINK1 kinase on the mitochondrial membrane, followed by the recruitment of Parkin, which tags mitochondria for degradation. P62 facilitates the transport of these mitochondria to the autophagosome and is itself degraded during active autophagy. Autophagosome formation requires Beclin1 and the conjugation of LC3-I to phosphatidylethanolamine, producing LC3-II, which is essential for autophagic progression

Mitochondria play a crucial role in both cellular and overall health. Various factors contributing to periodontitis can alter mitochondrial metabolic activity, release mtDNA, induce oxidative stress, and affect membrane dynamics (Yang et al. 2019). Mitochondria have long been recognized as the primary organelles responsible for energy metabolism in eukaryotic cells. As research into the MQC system advances, its complex functions are becoming increasingly clear (Hsu et al. 2013). The MQC system constitutes an integrated network that preserves mitochondrial homeostasis by regulating essential processes such as mitochondrial dynamics, mitophagy, and biogenesis (Fig. 3). These three core mechanisms—mitochondrial dynamics of the membrane, mitophagy, and mitochondrial normal biogenesis—collectively ensure the preservation of mitochondrial integrity and functionality.

### Mitochondrial dynamics

Mitochondrial dynamics play a crucial role in maintaining quality control and are integral to various mitochondrial functions. During their bioenergetic activities, mitochondria are exposed to numerous environmental fluctuations and stressors, both within and outside the cell, which can gradually impair their functionality. To mitigate this stress and enhance efficiency, mitochondria engage in fusion and exchange internal constituents (Roca-Portoles and Tait 2021). Mitochondrial dynamics refer to the balance between the fusion and fission processes, which govern mitochondrial morphology, function, and quantity. Mitochondrial fission is mainly regulated by Drp1 and Fis1. In contrast, fusion involves two distinct mechanisms: fusion of the outer mitochondrial membrane, controlled by Mfn1/2, and fusion of the inner mitochondrial membrane, led by OPA1 (Park et al. 2013).

### Mitophagy

Mitochondria can divide to remove damaged components accumulated over time, especially when the burden becomes excessive. The wasted components are subsequently decomposed into recyclable material by autophagy. Mitophagy is initiated by a decline in mitochondrial membrane potential, which results in the accumulation of the mitochondrial outer membrane kinase PINK1. This, in turn, recruits the E3 ubiquitin ligase Parkin from the cytoplasm (Abusleme and Moutsopoulos 2016). Following Parkin-mediated ubiquitination of mitochondria, these organelles are enveloped by an isolation membrane and fuse with lysosomes for degradation. Diverse variables can either impede or excessively augment mitophagy, leading to cellular malfunction or apoptosis (Yang et al. 2019; Pickles et al. 2018; Scaini et al. 2021).

### Mitochondrial biogenesis

Mitochondria cannot be synthesized entirely de novo; instead, their functionality is preserved through the dynamic exchange of older and newly formed components. PGC-1α serves as a critical regulator of mitochondrial biogenesis by facilitating the synthesis of TFAM and promoting mtDNA transcription via the activation of Nrf1/2 (Mehta et al. 2012; Valero 2014; Wang et al. 2011). Numerous antioxidants improve mitochondrial activity and safeguard cells by upregulating PGC-1α (Guak et al. 2022).

## Transcriptional alterations associated with mitochondrial and redox mechanisms in type 2 diabetes mellitus and periodontitis

Mitochondrial dysfunction is a key contributor to OS, manifesting as diminished antioxidant defenses and disruptions in transcriptional regulation. In individuals with concurrent T2DM-PD, poor glycemic control is linked to the accumulation of AGEs, an increase in red complex pathogens within the subgingival biofilm, and higher levels of pro-inflammatory mediators. Studies in both animal models and humans demonstrate that T2DM and periodontitis lead to mitochondrial dysfunction (Abusleme and Moutsopoulos 2016), characterized by reduced ATP synthesis, decreased mtDNA copy numbers, downregulation of complex I subunit expression, and heightened ROS production. Additionally, OS worsens the production of pro-inflammatory cytokines, such as TNF-α and IFN-γ, while impairing the activity of antioxidant enzymes like SOD, CAT, and GR. This decline in antioxidant capacity may be linked to altered transcription factors like Nrf2, HIF-1, and NF-κB, with elevated HIF-1α and NF-κB levels commonly observed in T2DM and periodontitis. Additionally, the regulation of antioxidant enzyme expression may be influenced by microRNAs, such as miR-223, which are implicated in T2DM and modulate the expression of antioxidants like HO-1, SOD1, and SOD2.

## The role of irradiation in periodontitis associated with DM

Mitochondrial dysfunction has emerged as a significant factor in the pathophysiology associated with DM, particularly in the irradiation exposure manifestations. Mitochondrial impairment can lead to a cascade of cellular dysfunctions. In patients with diabetes, mitochondrial dysfunction is exacerbated by oxidative stress, a condition that is further intensified by exposure to ionizing radiation. This relationship is particularly pertinent given that many cancer patients undergoing radiotherapy are also at risk for developing metabolic disorders, including diabetes (Montero and Patel. , 2015; Sroussi et al. 2017). Ionizing radiation induces the production of ROS, which can damage mtDNA more severely than nuclear DNA due to its proximity to the ETC where ROS are generated (Nuszkiewicz and Wo 2020). The persistent accumulation of OS and subsequent oxidative damage in mtDNA leads to mutations that impair mitochondrial function, resulting in decreased ATP production and increased apoptosis. This is particularly concerning in diabetic patients, where pre-existing mitochondrial dysfunction may sensitize cells to further damage from radiation. Studies have shown that irradiation can lead to a persistent reduction in mitochondrial content and functionality in a variety of cell types, such as endothelial cells, which are critical for vascular health (Bouten et al. 2021; Nuszkiewicz and Wo 2020). The implications of this are profound; as endothelial cells become dysfunctional due to radiation-induced mitochondrial impairment, they contribute to vascular complications often seen in diabetic patients, such as atherosclerosis and other cardiovascular diseases.

Furthermore, the interplay mechanisms between irradiation and mitochondrial dysfunction in DM is underscored by the role of mitophagy. In diabetic conditions, this process can be disrupted, leading to an accumulation of dysfunctional mitochondria that perpetuate oxidative stress and cellular injury (Ceballos-Picot et al. 1996). The failure of mitophagy not only exacerbates mitochondrial dysfunction but also contributes to the inflammatory processes that characterize diabetes and develop more progressive periodontitis. Additionally, the impact of radiation on mitochondrial morphology—such as changes in membrane potential and alterations in mitochondrial dynamics—has been observed and linked to impaired cellular metabolism and increased senescence. This is particularly relevant for diabetic patients who may already exhibit altered mitochondrial dynamics due to their metabolic state. The therapeutic implications of these findings are significant.

Understanding how irradiation affects mitochondrial function can inform strategies to mitigate these effects in diabetic patients undergoing cancer treatment. Pharmacological agents that enhance mitochondrial biogenesis or protect against oxidative stress may offer protective benefits against radiation-induced mitochondrial dysfunction. For instance, compounds like rosiglitazone have shown promise in preserving endothelial function post-irradiation by enhancing oxidative metabolism and reducing apoptosis. Such interventions could potentially reduce the risk of cardiovascular complications related to both diabetes and radiation therapy.

Moreover, research into the epigenetic effects of radiation on mitochondria reveals another layer of complexity. Radiation exposure can lead to fluctuations in gene expression related to mitochondrial function and metabolism through epigenetic modifications. These changes may not only affect immediate cellular responses but could also have long-term implications for metabolic health in cancer survivors with diabetes. The complex relationship between irradiation, mitochondrial dysfunction, and metabolic disease highlights the need for a multidisciplinary approach to patient care that considers both oncological and metabolic health.

## Clinical implications and therapeutic approaches

Mitochondrial dysfunction in diabetes mellitus represents a critical pathophysiological mechanism with far-reaching clinical implications. The compromised function of these cellular powerhouses leads to significant disturbances in energy production, manifesting as reduced ATP generation and impaired glucose utilization. This dysfunction creates a vicious cycle, where metabolic perturbations further exacerbate mitochondrial damage, contributing to the progression of diabetic complications. In pancreatic β-cells, mitochondrial dysfunction directly impacts insulin secretion, while in peripheral tissues, it promotes insulin resistance and OS. The clinical manifestations of mitochondrial dysfunction in diabetes present across multiple organ systems. In skeletal muscle, patients experience reduced exercise tolerance and compromised glucose uptake, contributing to overall metabolic inflexibility. Cardiac tissue demonstrates increased susceptibility to ischemia–reperfusion injury and reduced myocardial contractility, elevating cardiovascular risk. Neural tissue involvement accelerates the development of diabetic neuropathy and may contribute to cognitive decline. These tissue-specific effects collectively contribute to the complex clinical picture of diabetic complications.

Therapeutic approaches to addressing mitochondrial dysfunction in diabetes require a multi-faceted strategy. Lifestyle interventions form the foundation of treatment, with regular physical exercise playing a crucial role in enhancing mitochondrial biogenesis and function. Dietary modifications focus on antioxidant-rich foods and appropriate caloric intake complement exercise interventions. Stress management techniques are also incorporated to minimize oxidative stress and inflammation, which can further compromise mitochondrial function. Pharmacological interventions represent a crucial component of the therapeutic arsenal. Traditional antidiabetic medications like metformin demonstrate beneficial effects on mitochondrial function through AMPK activation. Thiazolidinediones improve mitochondrial biogenesis, while GLP-1 receptor agonists support overall mitochondrial health. Specific mitochondrial-targeted therapies, including Coenzyme Q10 supplementation, L-carnitine, and alpha-lipoic acid, provide additional support for mitochondrial function and antioxidant defense systems.

**Fig. 4:**
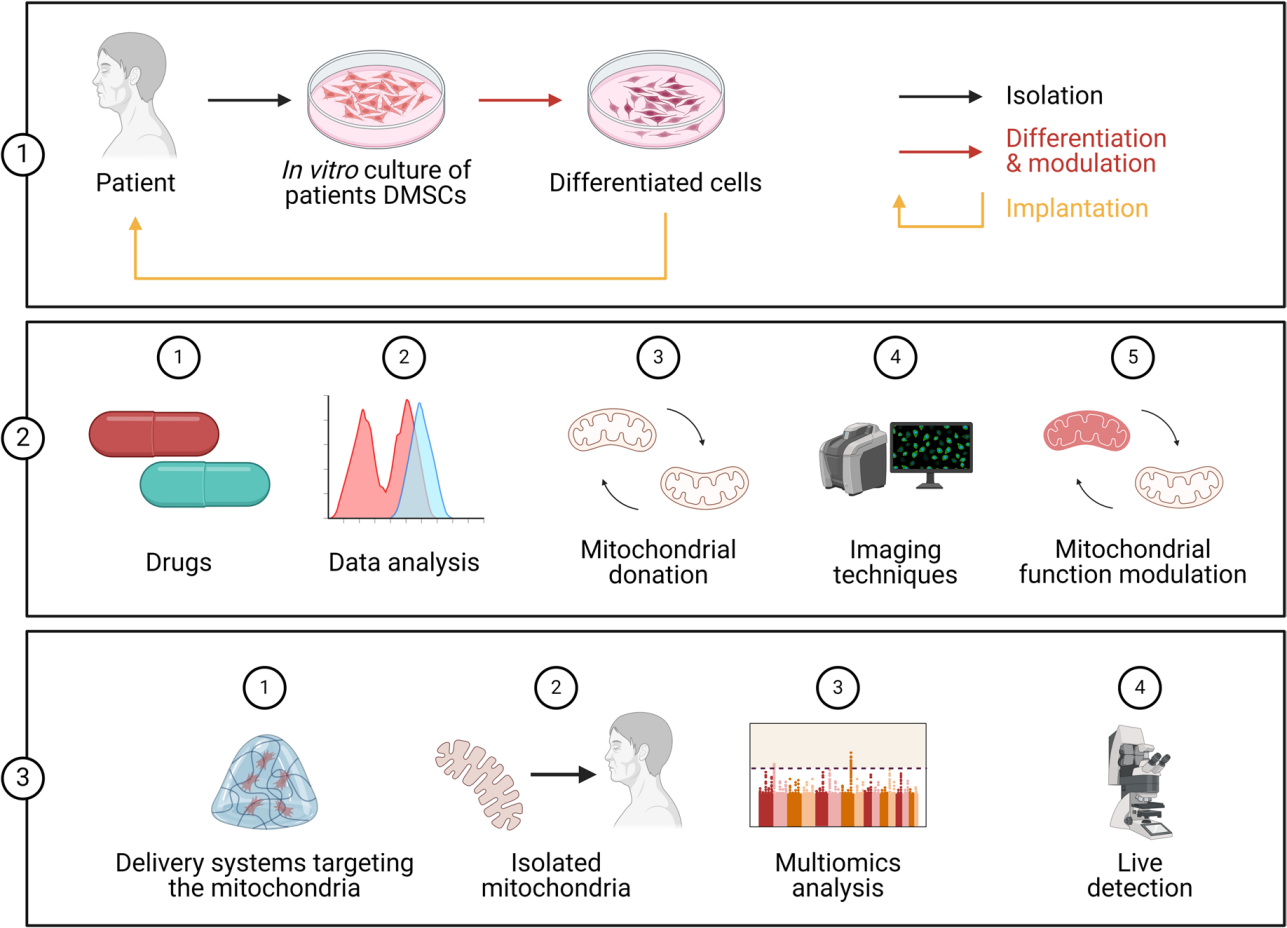
Current methodologies and emerging strategies in mitochondria-mediated regulation and therapeutic applications offer significant potential in the differentiation and development of DMSCs. Mitochondrial regulatory pathways and associated therapeutic interventions play a pivotal role in advancing regenerative medicine by modulating DMSC behavior. This paradigm involves isolating DMSCs from patients and engineering their mitochondrial functions to direct specific differentiation outcomes. These engineered DMSCs are subsequently utilized to repair or rejuvenate damaged or aged tissues and organs (1). Present-day approaches encompass pharmacological modulation, mitochondrial transfer via MSCs, advanced imaging modalities, data-driven analysis, and functional manipulation of mitochondrial activity (2). Prospective advancements aim to develop mitochondria-targeted drug delivery platforms, investigate the therapeutic potential of synthetic or isolated mitochondria, incorporate cutting-edge imaging and omics technologies for holistic mitochondrial profiling, and achieve targeted mitochondrial manipulation within a cellular and tissue-specific framework

Novel therapeutic strategies are emerging as our understanding of mitochondrial biology advances. Molecular approaches targeting key regulators like SIRT1 and PGC-1α show promise in enhancing mitochondrial function. Cell-based therapies, including stem cell treatments and mitochondrial transplantation, represent cutting-edge approaches currently under investigation (Fig. 4). Gene therapy targeting mitochondrial DNA offers the potential to address underlying genetic components of mitochondrial dysfunction. Clinical monitoring and management require a comprehensive approach. Regular assessment of glycemic control, organ function, and exercise capacity helps track treatment effectiveness. Laboratory testing for mitochondrial function markers, oxidative stress indicators, and metabolic parameters guide therapeutic adjustments (Soskic Branislava et al. 2011; Szliszka et al. 2009). This monitoring strategy ensures early detection of complications and allows for timely intervention modifications. Preventive strategies play a crucial role in managing mitochondrial dysfunction in diabetes. Early intervention in at-risk individuals, based on family history and clinical indicators, may help prevent or delay the onset of complications. Patient education focuses on lifestyle modification, medication adherence, and nutritional guidance and empowers individuals to actively participate in their treatment plan. Future therapeutic directions hold promises for improving patient outcomes (Soskic Branislava et al. 2011). Emerging technologies in drug development and delivery systems may provide more targeted approaches to treating mitochondrial dysfunction. Research focusing on mitochondrial-nuclear communication and epigenetic modifications continues to uncover new therapeutic targets. The integration of precision medicine approaches may allow for more personalized treatment strategies based on individual patient characteristics. Successful clinical management requires a comprehensive assessment and individualized treatment approach. Regular monitoring of treatment response and adjustment of interventions ensures optimal outcomes. Long-term follow-up care, including regular screening for complications and assessment of quality of life, remains essential for managing this chronic condition effectively.

The future of treating mitochondrial dysfunction in diabetes mellitus lies in the integration of multiple therapeutic modalities and the development of personalized treatment protocols. As our understanding of mitochondrial biology continues to expand, new therapeutic targets and treatment strategies will emerge. This evolution in treatment approaches, combined with early intervention and comprehensive management strategies, offers hope for improved outcomes in patients with diabetes-related mitochondrial dysfunction.

## Conclusion

In conclusion, the complex connections between mitochondrial dynamics, biogenesis, and mitophagy highlight their crucial roles in maintaining cellular homeostasis and responding to environmental challenges. The dysregulation of these processes is significantly implicated in the pathophysiology of conditions such as T2DM and Periodontitis, where oxidative stress and mitochondrial dysfunction converge to exacerbate disease progression. Understanding the mechanisms governing mitochondrial health offers promising approaches for therapeutic interventions to restore mitochondrial function and enhance cellular resilience. Moreover, the impact of external factors, such as irradiation, on mitochondrial integrity underscores the need for a comprehensive approach to address metabolic disorders in patients undergoing treatments for malignancies. Future research should continue to explore these relationships, aiming to elucidate further the molecular pathways involved and ultimately improve patient outcomes in conditions linked to mitochondrial dysfunction.

## Data Availability

All source data for this work (or generated in this study) are available upon reasonable request.
